# VIRAPOPS2 supports the influenza virus reassortments

**DOI:** 10.1186/1751-0473-9-18

**Published:** 2014-08-17

**Authors:** Michel Petitjean, Anne Vanet

**Affiliations:** 1 Univ Paris Diderot, Sorbonne Paris Cité, F-75013 Paris, France; 2 MTI, INSERM UMR-S 973, F-75013 Paris, France; 3 CNRS, UMR7592, Institut Jacques Monod, F-75013 Paris, France; 4 Atelier de Bio Informatique, F-75005 Paris, France

**Keywords:** Virology, Simulator, Population genetics, Segmented genomes

## Abstract

**Background:**

For over 400 years, due to the reassortment of their segmented genomes, influenza viruses evolve extremely quickly and cause devastating epidemics. This reassortment arises because two flu viruses can infect the same cell and therefore the new virions’ genomes will be composed of segment reassortments of the two parental strains. A treatment developed against parents could then be ineffective if the virions’ genomes are different enough from their parent’s genomes. It is therefore essential to simulate such reassortment phenomena to assess the risk of apparition of new flu strain.

**Findings:**

So we decided to upgrade the forward simulator VIRAPOPS, containing already the necessary options to handle non-segmented viral populations. This new version can mimic single or successive reassortments, in birds, humans and/or swines. Other options such as the ability to treat populations of positive or negative sense viral RNAs, were also added. Finally, we propose output options giving statistics of the results.

**Conclusion:**

In this paper we present a new version of VIRAPOPS which now manages the viral segment reassortments and the negative sense single strain RNA viruses, these two issues being the cause of serious public health problems.

## Introduction

**Figure 1:**
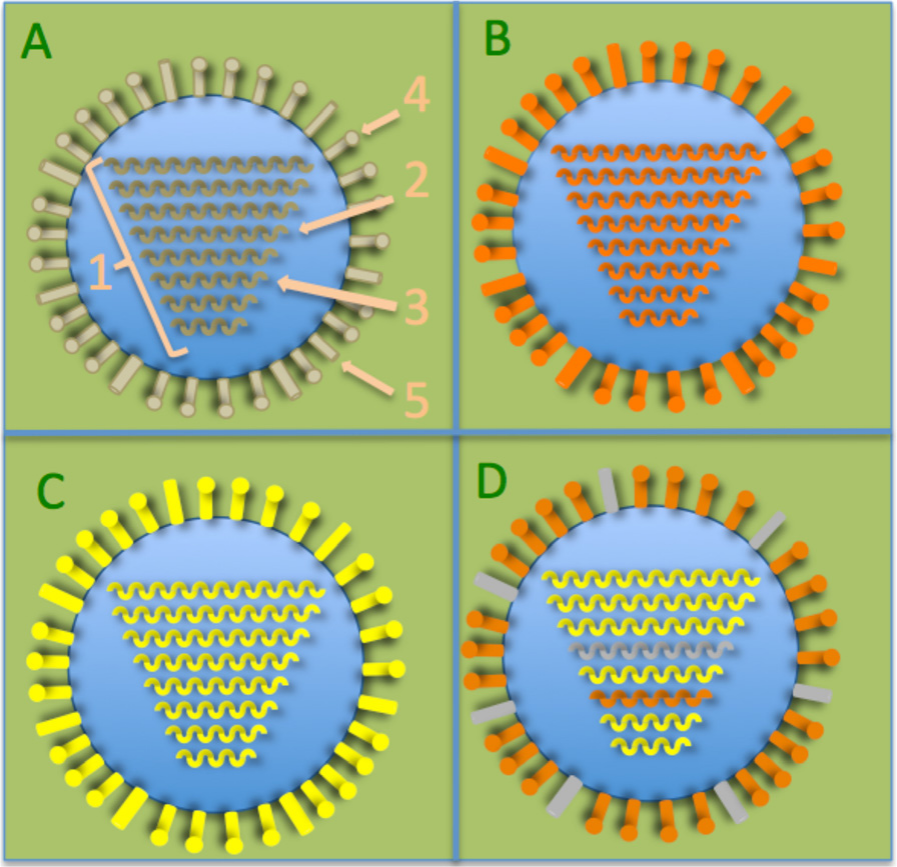
**Antigenic shift explains H7N9 emergence. A**. Scheme of flu virus. (gray color will be kept as meaningful for domestic duck). 1. 8 RNA segments of the viral genome. 2. Haemagglutinin (HA) coding segment. 3. Neuraminidase (NA) coding segment. 4. HA protein 5. NA protein. **B**. Wild bird flu virus. **C**. Domestic poultry flu virus. **D**. Sometimes, H7N9 virus made by avian RNA segment reassortment, can infect human. Note: the combination of the 16 H types and 9 N types can generate 144 different subtypes [9].

Each year worldwide, seasonal influenza causes between three and five million cases of severe illness and 250 000 to 500 000 deaths (OMS, 2014). Its variability explains this virulence: the low transcription fidelity and high recombination rate of these RNA viruses impose frequent genomic changes. Moreover, influenza viruses (Figure 1) possess a segmented genome (8 segments for type A and B and 7 segments for C) which further increases this variability. Indeed, the antigenic shift [1,2], phenomenon where two or more different influenza viruses (called parental viruses) may infect the same cell, can be responsible of devastating pandemics resulting from the creation of reassorted parental segment virions. To exemplify this fact, extremely virulent new strains such as H5N1 and H7N9 (Figure 1D and [3]) have appeared as the result of different reassortment processes.

Thus it becomes essential to be able to model these processes. On one hand, a large number of DNA population simulators [4] are not dedicated to RNA viruses that have specific mutational capacities such as compensatory mutations and synthetic lethal couples [5]. One the other hand, the small number of simulators specifically dedicated to virus, usually manages only the viral outbreaks [6]. These softwares do not simulate sequence population but rather evolution and number of infected individuals and other simulators handle the host-pathogen relationship [6]. Finally, the ‘reassortment and recombination’ simulator of Moedomo et al. [7] does not take into account the population genetic specific criteria. Yet the ‘reassortment and recombination’ feasibility depends directly on the viral fitness and therefore on selection pressure, population shift and drift. Ultimately the influenza simulators do not generate nucleic or proteic sequences and the genetic population simulators are not endowed with necessary options for RNA viruses.

### VIRAPOPS

The viral RNA mutability allows rapid selection of drug resistances: some active viral proteins may have up to 30% of their positions mutated. The conventional population simulators are not able to model selection pressures on groups of mutations genetically linked. To stage these different scenarios we have developed VIRAPOPS a forward simulator dedicated to RNA viruses, easy to use and answering questions related to these highly specific populations. Despite that VIRAPOPS simulates viral genetic variability through a number of functionalities, it lacks a significant functionality to simulate flu populations: it was unable to handle the reassortment of segmented genomes, essential phenomenon in public health (see Introduction), and negative strand RNA viruses (such as Ebola and measles among others). Therefore, we decided to enrich VIRAPOPS [8], which possesses good foundations to integrate these new options. Additionally, new output options generate statistics on the obtained sequence sets.

### Mathematical models

The negative strand model is derived as follows. Let *E* be the set of allowed symbols in the sequences and Φ a bijection mapping *E* onto *E*, Φ^−1^ exists and is such that ∀ *x* ∈ *E*, *Φ*(*Φ*^− 1^(*x*)) = *x* and *Φ*^− 1^(*Φ*(*x*)) = *x*. Furthermore, Φ is required to be a *derangement*, i.e. the subset of *E* such that *Φ*(*x*) = *x* must be void.

Here we have *E* = {*A*, *C*, *G*, *T*}. There is 9 possible derangements: 3 circular permutations with 1 cycle of order 4 plus their 3 inverse permutations (also circular with 1 cycle of order 4), and 3 symmetric permutations (i.e. *Φ*^− 1^ = *Φ*) containing 2 cycles of order 2. Our negative strand model is defined with such a symmetric permutation: *Φ*(*A*) = *T*, *Φ*(*T*) = *A*, *Φ*(*C*) = *G*, *Φ*(*G*) = *C*.

The reassortment model has two parts. The first part of the model is an uniform sampling without replacement. Let *N* be the total number of sequences and *n* be the number of sequences to be reassorted. There are $\frac{N!}{n!\left(N-n\right)!}$ subsets of *n* sequences among *N*. Thus the first part of the model is defined by a random selection of these subsets of *n* sequences, all the subsets being equiprobable.

The second part of the model is a random shuffling. Let *K* be the number of fragments. For each fragment *k* ∈ {1, 2,...., *K*}, there are *n* subsequences. A random permutation *P*_*k*_ of order *n* is generated with a user defined probability depending on *k*, 1 ≤ *k* ≤ *K*, following the uniform law in the space of the *n!* permutations. Then each *P*_*k*_ is applied to the *n* subsequences.

Here we have set *n = 2* with planned extensions to higher *n* values, and usually *K* = 7 or *K* = 8.

This option can simulate the influenza virus reassortments, but also all other segmented virus reassortments, whatever the number of segments. Thus VIRAPOPS2 may simulate rotavirus populations responsible for acute gastroenteritis whose genetic variability is also due to antigenic shift of its 11 segments [9].

Free binaries and documentations are available through a software repository at http://petitjeanmichel.free.fr/itoweb.petitjean.freeware.html.

### Statistics

The sequences of the population individuals derived from the simulation was the only output of the previous version of VIRAPOPS. In order to analyze this sequence set, users had to rely on other software. New VIRAPOPS2 output options allow the determination of the number of mutation per position (DNA and protein), the most frequent mutations per position (DNA and protein) and finally the consensus sequences (DNA and protein).

### Negative and positive sense viral RNA

A negative single strand RNA (also called minus RNA) codes numerous viruses, as measles, rabies, mumps and Ebola. The previous version of VIRAPOPS only allowed the simulation of positive RNA viruses. In this version we added a new option where the user has to choose the sense of the studied RNA.

### Example of use

#### Flu from bird to man

Public health agencies want to slow emergence of new influenza strains coming from avian virus rearrangements [10,11], e.g. the H7N9 virus which seems to come from rearrangement of domestic ducks, wild birds and domestic poultry viruses [12]. VIRAPOPS2 can predict the occurrence of such rearranged viruses (Figure 1) and we propose to simulate this scenario using a flu viral sequence from domestic duck to infect a seronegative bird. The functional selection pressures (option 10 and 11) and recombination (option 9) will be reproduced as explain in Petitjean and Vanet [8] documentation. The simulator can be launched on 80 generations (option 3) with 50 budded virions per cycle (option 4). Then at the 40th generation, the gene flow option (#14) allows us to simulate the infection of the bird with wild bird and domestic poultry viruses. Genetic shift option (#9) permits to model the reassortment process with a different probability for each segment and will be active until the end of the simulation. If a virus issued from these 3 avian viral reassortments appears, it will be part of VIRAPOPS2 output.

#### Treatment and antigenic shift

An effective treatment applies a selection pressure on a particular virus. When viruses evolve quickly, the impact of treatment on viral populations is unknown. Therefore yearly a new vaccine against seasonal influenza has to be developed. To solve such problem, the simulation of the influenza virus rearrangement (equivalent to previous setting) will has to be compared with a new simulation in which the treatment selection pressures (option 12) will be included (e.g. H275Y is a neuraminidase resistance mutation allowing the influenza viruses to escape oseltamivir treatment) [13]. The comparison of the two resulting sequence sets can determine the treatment impact on rearranged viruses.

## Conclusion

It is essential to describe in the same simulator all the criteria that influence the population evolution as recombination, mutational variability, selection pressures, population drifts and shifts. These options will allow the description of the viral diversity in a given environment and the ability to identify the virus that has the best fitness and therefore the most represented in the population. It is through these fitness determinations that the simulator will highlight viruses that have the best chance of survival and the possibility to invade the whole population. These viruses have to be detected because they will cause the next epidemic.

## Competing interest

The authors declare that they have no competing interests.

## Authors’ contributions

MP has built the antigenic shift program. AV has conceived the design of the study and wrote the manuscript. Both authors read and approved the final manuscript.
